# Efficacy and safety of first-line immunotherapy-related induction and maintenance therapy for extensive-stage small cell lung cancer: a systematic review and Bayesian network meta-analysis

**DOI:** 10.3389/fphar.2026.1944817

**Published:** 2026-09-15

**Authors:** Hong-Jing Yu, Jun-Chen Liu, Dong-Sheng Zhang, Zhi Chen, Qi Deng

**Affiliations:** 1 Department of Oncology, The First People’s Hospital of Jiande, Jiande, China; 2 Department of Clinical Pharmacy, The First People’s Hospital of Jiande, Jiande, China; 3 Department of General Surgery, The First People’s Hospital of Jiande, Jiande, China; 4 Department of Clinical Pharmacy, The First People’s Hospital of Jiande, Jiande, China

**Keywords:** Bayesian network meta-analysis, efficacy and safety, extensive-stage small cell lung cancer, first-line therapy, immune checkpoint inhibitors

## Abstract

**Background:**

Extensive-stage small cell lung cancer (ES-SCLC) is highly aggressive with dismal prognosis, and platinum monochemotherapy offers modest long-term survival. Multiple first-line regimens integrating immunotherapy, anti-angiogenic agents and chemotherapy have been approved, yet direct head-to-head comparisons are scarce, and the optimal induction plus maintenance strategy remains undefined. In this study, a Bayesian network meta-analysis (NMA) was performed to hierarchically assess the efficacy and safety of available first-line regimens and identify balanced treatment strategies for ES-SCLC.

**Methods:**

Eligible phase III randomized controlled trials (RCTs) were identified by searching PubMed, Embase, the Cochrane Library, and Web of Science. Trials were included if they enrolled treatment-naïve patients with ES-SCLC and reported at least one of the following outcomes: overall survival (OS), progression-free survival (PFS), or grade ≥3 treatment-related adverse events (TRAEs). NMA was implemented using the gemtc package in R. SUCRA values were calculated for treatment ranking, and subgroup analyses stratified by pharmacological mechanisms were conducted.

**Results:**

Fourteen RCTs involving 8,945 patients across 18 regimens were included in the present analysis. These trials encompassed diverse combinations of mainstream immune checkpoint inhibitors, anti-angiogenic targeted agents, and chemotherapeutic agents. The atezolizumab + lurbinectedin + chemotherapy and benmelstobart + anlotinib + chemotherapy regimens showed the greatest potential for superior PFS and OS compared with chemotherapy alone. Subgroup analyses confirmed sustained efficacy benefits for PD-L1 inhibitor-based triple-combination strategies. The nivolumab + ipilimumab regimen was associated with the highest risk of severe toxicity.

**Conclusion:**

PD-L1 inhibitor combined with platinum-based chemotherapy together with anti-angiogenic agents or lurbinectedin conferred optimal survival benefits, yet toxicity was correspondingly augmented. This study provides valuable insights into therapeutic drug selection for ES-SCLC.

**Systematic Review Registration:**

https://www.crd.york.ac.uk/PROSPERO/view/CRD420261402898, identifier 420261402898.

## 1 Introduction

Small cell lung cancer (SCLC) represents the most aggressive subtype of lung cancer, accounting for approximately 15% of all lung malignancies (Gazdar et al., 2017). ES-SCLC accounts for around 70% of newly diagnosed SCLC cases and is characterized by early distant metastasis, rapid tumor proliferation, and a high propensity to develop chemotherapy resistance (van Meerbeeck et al., 2011). Prior to 2019, etoposide plus platinum doublet chemotherapy served as the global standard first-line regimen for ES-SCLC (Socinski et al., 2009; Rossi et al., 2012). Nevertheless, patients achieved a median OS of only 10 months; over 80% experienced rapid disease progression within 6 months, and the 5-year survival rate reached merely 7% (Byers and Rudin, 2015). There is an urgent clinical need for novel therapeutic strategies to improve the long-term survival of these patients.

Immune checkpoint inhibitors targeting PD-1/PD-L1 have broken the therapeutic deadlock of ES-SCLC. Two landmark phase III trials, IMpower133 and CASPIAN, first validated that adding atezolizumab or durvalumab to platinum-based chemotherapy significantly prolonged overall survival, establishing PD-L1 inhibitor combined with chemotherapy as the updated standard first-line regimen (Horn et al., 2018; Paz-Ares et al., 2019). Since then, multiple PD-1 inhibitors (nivolumab, serplulimab, adebrelimab, toripalimab), anti-angiogenic agents (bevacizumab, anlotinib), CTLA-4 antibodies (ipilimumab, tremelimumab), and the novel agent lurbinectedin have been incorporated into induction or maintenance treatment frameworks. These combinations have generated dozens of combinatorial regimens with markedly distinct efficacy and safety profiles (Reck et al., 2016; Rudin et al., 2020; Owonikoko et al., 2021; Cheng et al., 2022; Wang et al., 2022; Cheng et al., 2024a; Cheng et al., 2024b; Ohe et al., 2024; Rudin et al., 2024; Chen et al., 2025; Cheng et al., 2025; Paz-Ares et al., 2025a).

However, multiple critical clinical controversies remain to be resolved. First, head-to-head randomized controlled trials comparing various immunotherapy combination regimens are lacking, and inconsistent eligibility criteria and follow-up durations across existing studies prevent clinicians from directly identifying the optimal induction plus maintenance regimen. Second, there is a lack of quantitative stratified evaluation balancing anti-tumor efficacy and the risk of severe toxicities. Triple combinations of immunotherapy, chemotherapy and anti-angiogenic agents exert stronger anti-tumor activity yet may lead to overlapping treatment-related adverse events; by contrast, single-agent immunotherapy or simplified dual-drug regimens carry milder toxicities but yield compromised survival benefits (Saida et al., 2023; Cheng et al., 2024a). Third, evidence stratified by the mechanism of drug action is insufficient, which hinders the development of individualized therapeutic strategies for specific patient subgroups such as the elderly and those with poor organ function tolerance.

NMA enables integration of direct and indirect comparative evidence from multiple randomized controlled trials. It allows hierarchical ranking of diverse interventions simultaneously across multiple clinical endpoints, overcoming the limitations of conventional pairwise meta-analysis (Lu and Ades, 2004). This study seeks to compare OS, PFS, and TRAEs among mainstream first-line immunotherapy-containing regimens for ES-SCLC, characterize the benefit-risk balance of distinct therapeutic strategies, and provide evidence that may inform stratified treatment suggestions to facilitate individualized clinical decision-making. To this end, we systematically collected all published RCTs investigating first-line immunotherapy induction and maintenance regimens for ES-SCLC. A Bayesian NMA was performed, and surface under the cumulative ranking curve (SUCRA) values were used to rank all regimens according to OS, PFS, and TRAEs; subgroup analyses stratified by drug classes were further conducted.

## 2 Materials and methods

In this study, eligible phase III first-line ES-SCLC trials were categorized by protocol-specified treatment sequences. Patients were randomized at first-line enrolment into two patterns: (1) Induction-followed-by-maintenance regimens: patients received intensive induction combination therapy; non-progressing patients entered a pre-defined maintenance phase with scheduled study-drug administration, including active-drug or placebo maintenance for control arms in double-blinded trials. (2) Induction-only regimens: no protocol-defined maintenance-phase medication (active agent or placebo) was given after induction, with only routine follow-up; this group mainly consisted of control arms. Notably, CheckMate 451 had a distinct design: randomization and survival-endpoint assessment occurred post-induction among non-progressing patients, not at initial first-line enrolment. A sensitivity analysis excluding CheckMate 451 was performed to address this discrepancy in estimand and time-zero definition. Each treatment node represented a complete first-line therapeutic sequence. Outcome evaluation started at initial randomization of treatment-naïve ES-SCLC patients for nearly all trials. Therefore, this network meta-analysis compared overall efficacy and safety of holistic first-line strategies rather than isolated maintenance-phase effects.

### 2.1 Search strategy

Comprehensive literature retrieval was performed across the PubMed, Embase, Cochrane Library, and Web of Science databases from database inception to 30 May 2026. The search strategy combined medical subject headings and free-text terms: (extensive-stage small cell lung cancer OR ES-SCLC) AND (immune checkpoint inhibitor OR PD-1 OR PD-L1 OR CTLA-4 OR atezolizumab OR durvalumab OR pembrolizumab OR nivolumab OR ipilimumab OR tiragolumab OR sintilimab OR toripalimab OR camrelizumab OR tislelizumab OR adebrelimab OR serplulimab OR socazolimab OR bevacizumab OR anlotinib) AND (first-line OR induction OR maintenance) AND (randomized controlled trial) (Supplementary Table S1). Manual screening of the reference lists of eligible original studies, systematic reviews, and clinical guidelines was conducted to supplement potentially qualified literatures, with no restrictions on publication language.

### 2.2 Eligibility criteria

#### 2.2.1 Inclusion criteria

Studies eligible for inclusion met the following criteria: (1) Study design: phase III randomized controlled trials (RCTs); (2) Study population: patients pathologically diagnosed with ES-SCLC and without prior systemic anti-tumor therapy; (3) Interventions: experimental arms adopted PD-1/PD-L1/CTLA-4 inhibitor combined with chemotherapy, anti-angiogenic agents or maintenance therapy, while control arms received platinum-only chemotherapy or alternative immunotherapy combination regimens; (4) Outcome indicators: studies reporting at least one primary endpoint of OS, PFS, or TRAEs, with extractable hazard ratios (HRs) or odds ratios (ORs) and corresponding 95% confidence intervals (CIs).

#### 2.2.2 Exclusion criteria

Studies were excluded based on the following criteria: retrospective cohort studies, single-arm trials, case reports, reviews, and meta-analyses were excluded. Trials enrolling patients with limited-stage small cell lung cancer or receiving second-line or subsequent treatment were excluded. Studies lacking available quantitative survival and safety data for network comparison modeling were excluded. Overlapping data from repeatedly published reports of the same clinical trial were also excluded.

## 3 Data extraction and risk of bias assessment

Literature screening and data extraction were performed independently by two investigators. Discrepancies were resolved through group discussion to reach a consensus. Extracted information covered trial name, publication year, sample size, baseline patient characteristics, treatment regimens, median follow-up duration, and quantitative data of OS, PFS and grade ≥3 AEs.

The Cochrane Risk of Bias Tool 2.0 (ROB 2.0) was utilized to assess the risk of bias of included RCTs in strict accordance with the recommended specifications of the Cochrane Handbook for Systematic Reviews of Interventions. The assessment domains consisted of random sequence generation, allocation concealment, blinding of participants and researchers, completeness of outcome data, selective outcome reporting, and other potential sources of bias.

## 4 Statistical analysis

All statistical analyses for NMA were performed using R4.3.1 software with the gemtc package. A network comparison dataset was constructed via the mtc.network() function, and a fixed-effect consistency model was established using mtc.model(). The model was specified with a Poisson likelihood function and a log link function, and four independent Markov chain Monte Carlo (MCMC) sampling chains were automatically generated by the program. For MCMC sampling, 20,000 adaptive iterations were first run to optimize sampling parameters, followed by 50,000 formal iterations to obtain posterior distributions with a thinning interval (thin) set to 1. Model convergence was confirmed when the potential scale reduction factor (PSRF) was less than 1.05, complying with standardized methodological requirements for NMA in oncology research (Neupane et al., 2014). Fixed-effects consistency model was used in this NMA to guarantee the model’s robustness. The deviance information criterion (DIC) was calculated to compare and evaluate the fitness of fixed-effect and random-effect models (Dias et al., 2010). Trace plots were visualized, and Brooks–Gelman–Rubin statistics were computed to examine adequate model convergence, defined as the attainment of a stable equilibrium distribution (Brooks and Gelman, 1998).

Treatment regimens were ranked quantitatively based on the SUCRA values (Salanti et al., 2011). For OS and PFS endpoints, higher SUCRA values indicated superior survival benefit and tumor progression inhibition. For TRAEs, higher SUCRA values represented a lower risk of severe treatment-related toxicity and better safety profile.

Subgroup analyses stratified by therapeutic mechanism were conducted to verify the robustness of ranking results. All included regimens were categorized into eight groups as follows: PD-L1 inhibitor plus platinum-based chemotherapy (CT) induction followed by lurbinectedin maintenance (PDL1+CT+Lur), PD-L1 inhibitor plus anti-angiogenic agents (AA) plus chemotherapy (PDL1+AA+CT), PD-L1 inhibitor combined with chemotherapy (PDL1+CT), PD-1 inhibitor combined with chemotherapy (PD1+CT), single-agent PD-1 inhibitor (PD1), dual immunotherapy combined with chemotherapy (PDL1+CTLA4+CT/Tira+PDL1+CT), anti-angiogenic agents combined with chemotherapy (AA+CT), and platinum-only chemotherapy.

## 5 Results

### 5.1 Basic characteristics of included studies

After multi-round screening, a total of 14 phase III RCTs including IMpower133, CASPIAN, CheckMate451, IMforte, ETER701, RATIOOLE-312, ASTRUM-005, SKYSCRAPER-02, CAPSTONE-1 and ZKAB001 were finally enrolled. Eighteen distinct first-line treatment regimens were incorporated into the NMA (Figure 1; Table 1).

**FIGURE 1:**
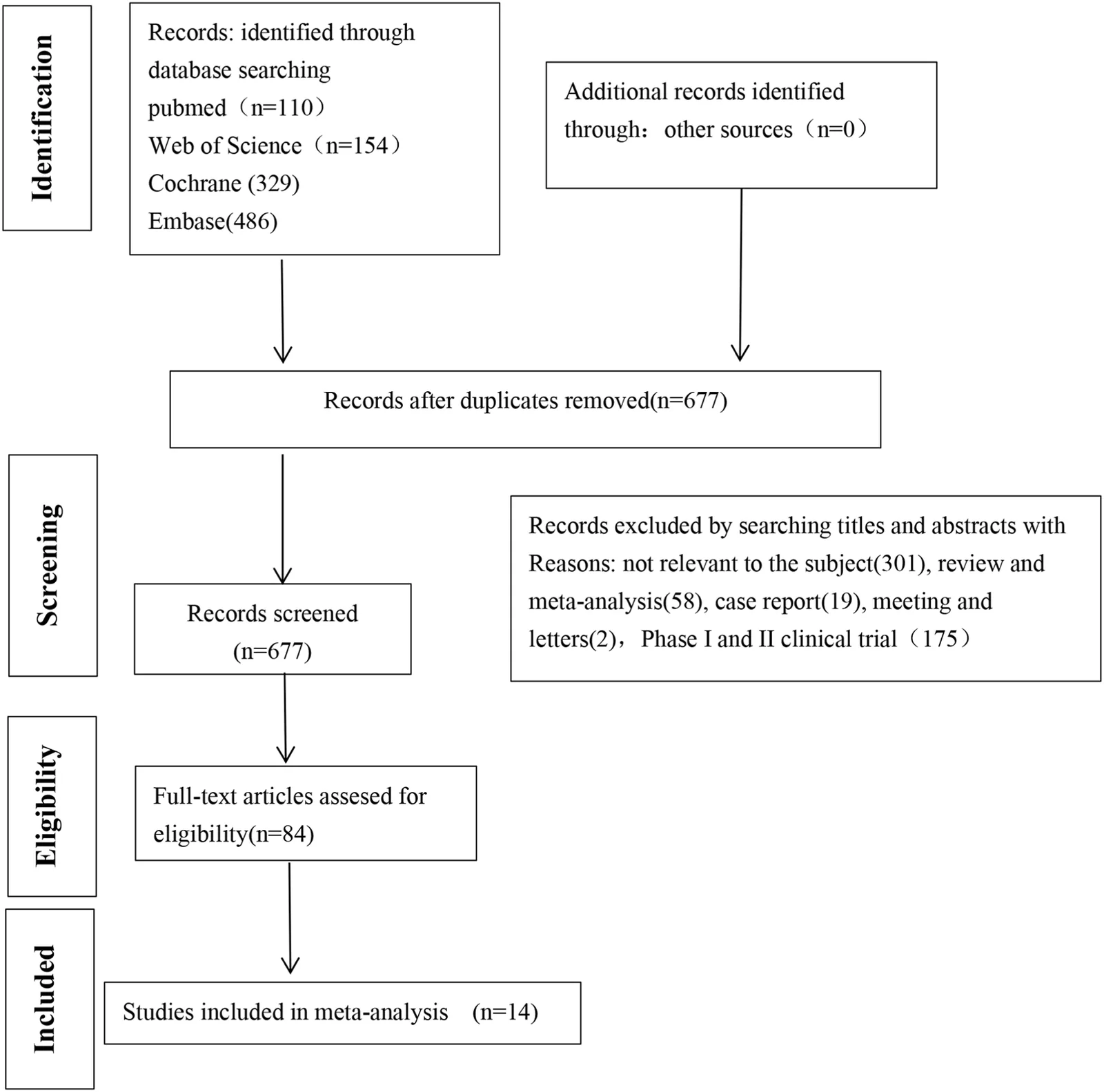
Flow diagram of meta-analysis for inclusion/exclusion of studies.

**TABLE 1:** The baseline characteristics of included trials.

No.	Study	Author	Year	Intervention arm	No. of patients	Control arm	No. of patients	OS (HR, 95%CI)	PFS (HR, 95%CI)
1	Impower133	Stephen et al.	2018	Ate+CT→Ate maintenance	201	Placebo+CT→ Placebo maintenance	202	0.76 (0.60, 0.95)	0.77 (0.62, 0.92)
2	CheckMate 451	Owonikoko et al.	2021	CT→Nivo maintenance	280	CT→Placebo maintenance	275	0.84 (0.69, 1.02)	0.67 (0.56, 0.81)
3	CheckMate 451	Owonikoko et al.	2021	CT→Nivo+Ipi maintenance	279	CT→Placebo maintenance	275	0.92 (0.75, 1.12)	0.72 (0.60, 0.87)
4	IMforte	Paz,Ares L et al.	2025	Ate+CT→Ate+Lur maintenance	242	Ate+CT→Ate maintenance	241	0.73 (0.57, 0.95)	0.54 (0.43, 0.67)
5	ETER701	Cheng et al.	2023	Ben+Anlo+CT→Ben+Anlo maintenance	246	Placebo+CT→ Placebo maintenance	247	0.61 (0.47, 0.79)	0.32 (0.26, 0.41)
6	ETER701	Cheng et al.	2023	Anlo+CT→Anlo maintenance	245	Placebo+CT→ Placebo maintenance	247	0.86 (0.67, 1.10)	0.44 (0.36, 0.55)
7	RATIONALE-312	Cheng et al.	2023	Tis+CT→Tis maintenance	227	Placebo+CT→ Placebo maintenance	230	0.75 (0.61, 0.93)	0.64 (0.52, 0.78)
8	CASPIAN	Goldman et al.	2019	Dur+CT→Dur maintenance	268	CT	269	0.71 (0.60, 0.86)	0.80 (0.66, 0.96)
9	CASPIAN	Goldman et al.	2019	Dur+Tre+CT→Dur maintenance	268	CT	269	0.81 (0.67, 0.97)	0.84 (0.70, 1.01)
10	ASTRUM-005	Cheng et al.	2022	Ser+CT→Ser maintenance	389	CT→Placebo maintenance	196	0.62 (0.50, 0.76)	0.47 (0.38, 0.58)
11	SKYSCRAPER-02	Rudin et al.	2022	Tira+Ate+CT→Tira+Ate maintenance	243	Ate+CT→ Placebo+Ate maintenance	247	1.09 (0.88, 1.35)	1.08 (0.89, 1.31)
12	CAPSTONE-1	Wang et al.	2022	Ade+CT→Ade maintenance	230	Placebo+CT→ Placebo maintenance	232	0.72 (0.58, 0.90)	0.67 (0.54, 0.83)
13	ZKAB001	Lu et al.	2025	ZKA+CT→ZKA maintenance	248	Placebo+CT→ Placebo maintenance	248	0.80 (0.65, 0.98)	0.57 (0.46, 0.71)
14	BEAT-SC	Ohe et al.	2024	Bev+Ate+CT→Bev+Ate maintenance	167	Placebo+Ate+CT→ Placebo+Ate maintenance	166	1.22 (0.89, 1.67)	0.70 (0.54, 0.90)
15	KEYNOTE-604	Rudin et al.	2020	Pem+CT→Pem maintenance	228	Placebo+CT →Placebo maintenance	225	0.80 (0.64, 0.98)	0.75 (0.61, 0.91)
16	EXTENTORCH	Cheng et al.	2023	Tor+CT→Tor maintenance	223	Placebo+CT→ Placebo maintenance	219	0.80 (0.65, 0.98)	0.67 (0.54, 0.82)
17	CA184-156	Reck et al.	2016	Ipi+CT→Ipi maintenance	478	Placebo+CT→ Placebo maintenance	476	0.94 (0.81, 1.09)	0.85 (0.75, 0.97)

Abbreviations: Ade, adebrelimab; Anlo, anlotinib; Ate, atezolizumab; Ben, benmelstobart; Bev, bevacizumab; CI, confidence interval; CT, chemotherapy; Dur, durvalumab; HR, hazard ratio; Ipi, ipilimumab; Lur, lurbinectedin; Nivo, nivolumab; Pem, pembrolizumab; Ser, serplulimab; Tis, tislelizumab; Tira, tiragolumab; Tor, toripalimab; Tre, tremelimumab; ZKA, socazolimab.

Risk of bias assessment using the ROB 2.0 tool indicated an overall low-to-moderate risk of bias across all included trials. Complete reporting of primary efficacy and safety endpoints guaranteed the reliability of subsequent quantitative pooled results (Supplementary Figure S1). Trace plots and density plots were presented in Supplementary Figure S6, respectively. The network comparison graph demonstrated satisfactory connectivity across all regimens without isolated nodes, supporting the feasibility of indirect comparisons (Figure 2).

**FIGURE 2:**
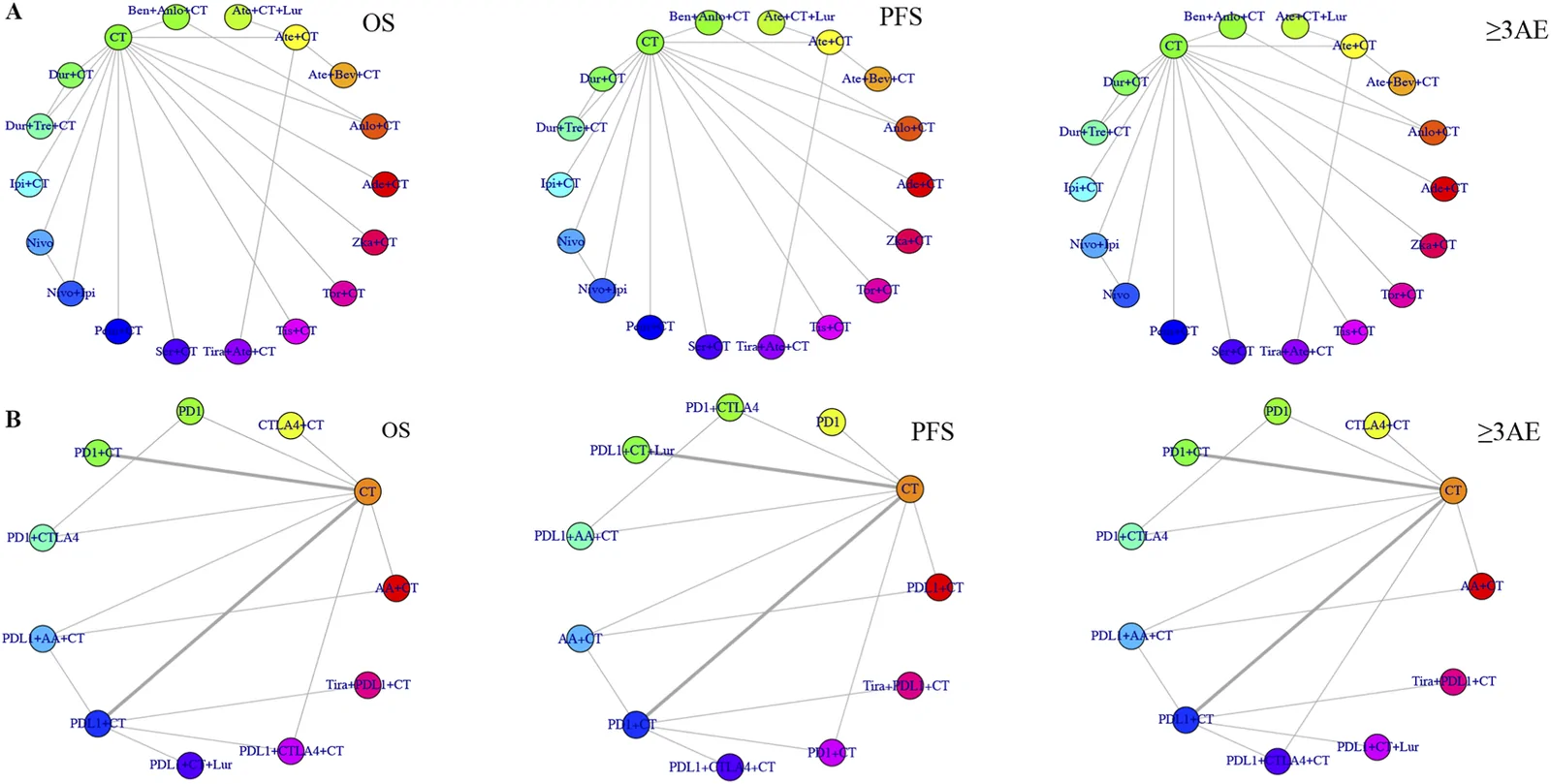
NMA of comparisons of different outcomes of first-line treatments in various groups of ES-SCLC patients. **(A)** All therapeutic regimens in the overall patient cohort. **(B)** Pharmacological classification subgroup analysis. Each circle represents a treatment, and the line between the two points represents a comparison between the two treatments.

### 5.2 Network meta-analysis (NMA)

#### 5.2.1 PFS and OS

For PFS, NMA demonstrated that most immunotherapy-based regimens significantly reduced the risk of tumor progression relative to platinum-only chemotherapy (CT). The hazard ratios (HRs) of Ate+CT+Lur, Ben+Anlo+CT, Ser+CT, Tis+CT, Nivo, Tor+CT, Zka+CT, Ade+CT, Nivo+Ipi, Pem+CT, Ate+CT, Dur+CT and Ate+Bev+CT versus CT were all less than 1, with corresponding 95% credible intervals (CrIs) not crossing 1. Only two regimens exhibited non-significant differences versus CT: Dur+Tre+CT (HR = 0.84, 95% CrI: 0.70–1.01) and Tira+Ate+CT (HR = 0.83, 95% CrI: 0.63–1.09), whose 95% CrIs crossed the null value of 1. Among all immunotherapy-containing regimens, Ben+Anlo+CT yielded substantially prolonged PFS compared with almost all alternative treatments (all HR < 1, 95% CrI not crossing 1), with the sole exception of Ate+CT+Lur (HR = 0.77, 95% CrI: 0.50–1.05). Other regimens with prominent progression-suppressing benefits included Anlo+CT, Ate+CT+Lur and Ser+CT (Figure 3B).

**FIGURE 3:**
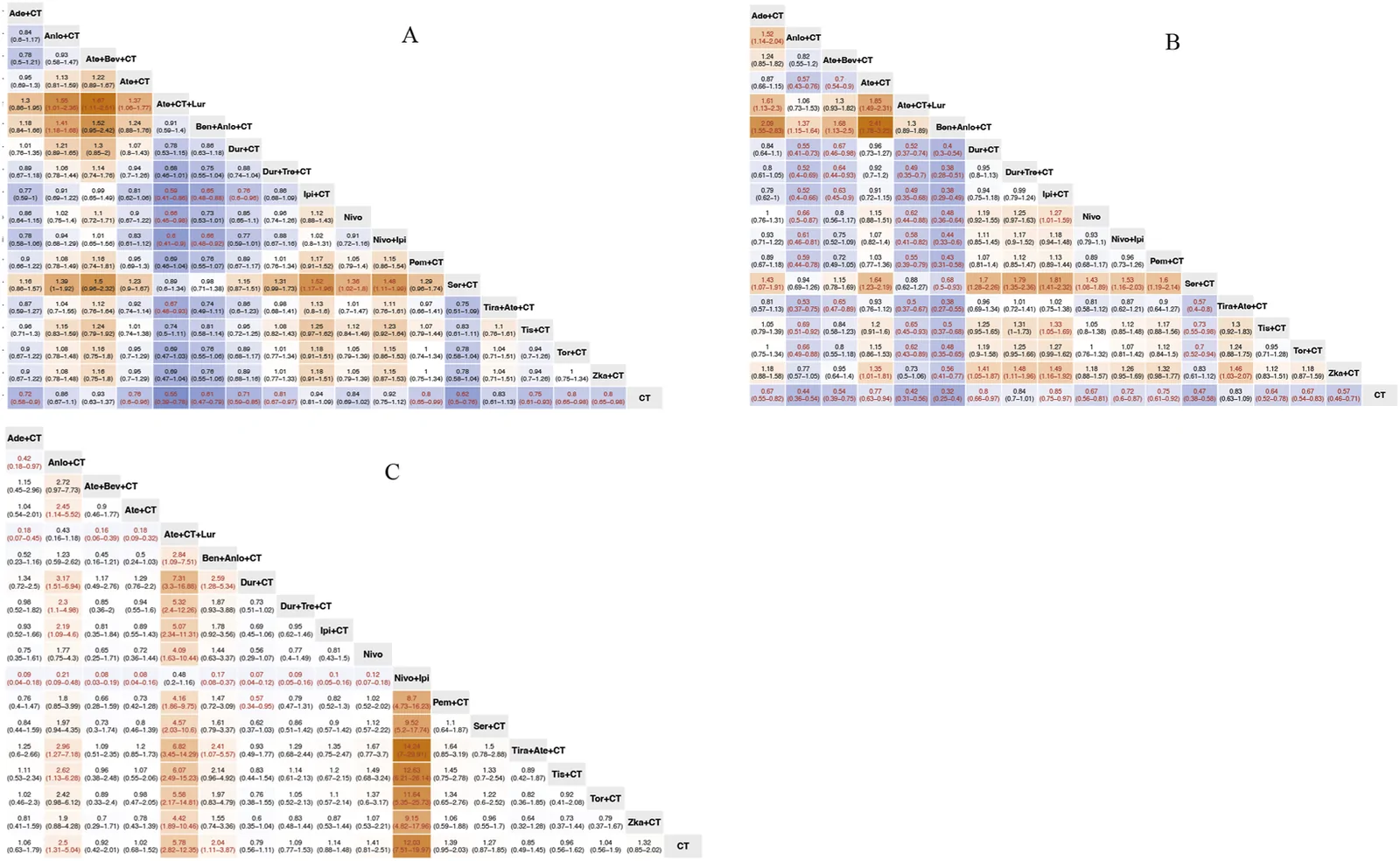
Efficacy and safety summaries from NMA in ES-SCLC patients: **(A)** OS, **(B)** PFS, and **(C)** AEs. The data in each cell represent the HR or OR values that compare treatment effects as defined by the columns and rows. The cells are color-coded according to the HR or OR values: values less than 1 are shaded blue, values greater than 1 are shaded orange, and cells with a value equal to 1 or missing data are filled with white. The important HRs (or ORs) and 95% CrIs are highlighted in red.

With regard to OS, consistent findings were observed with PFS: the majority of immunotherapy-based regimens were superior to CT, with all HR values less than 1 and most 95% CrIs excluding the null value of 1. Ate+CT+Lur exerted the most prominent survival benefit (HR = 0.55, 95% CrI: 0.39–0.78), followed by Ben+Anlo+CT (HR = 0.61, 95% CrI: 0.47–0.79) and Ser+CT (HR = 0.62, 95% CrI: 0.50–0.76). Statistically significant superiority of Ate+CT+Lur was also identified when compared with most other treatment regimens (Figure 3A).

#### 5.2.2 Grade ≥3 adverse events

All 14 included RCTs were eligible for NMA of grade ≥3AEs (Figure 3C). Four immunotherapy-containing regimens presented significantly higher risks of grade ≥3 AEs relative to CT, namely Nivo+Ipi (OR = 12.03, 95% CrI: 7.51–19.97), Anlo+CT (OR = 2.50, 95% CrI: 1.31–5.04), Ate+CT+Lur (OR = 5.78, 95% CrI: 2.82–12.35) and Ben+Anlo+CT (OR = 2.04, 95% CrI: 1.11–3.87). Notably, Nivo+Ipi was associated with markedly elevated risks of severe AEs compared with all other immunotherapy-based regimens, with all corresponding ORs and 95% CrIs excluding the null value of 1. Triple-agent combinations yielded substantially increased toxic burden upon pairwise comparisons against alternative regimens. Ate+CT+Lur significantly raised the incidence of grade ≥3 AEs, whereas overall toxicity remained manageable for Ben+Anlo+CT. No significant elevation in the risk of grade ≥3 AEs was detected for Ser+CT, either versus other immunotherapy regimens or platinum-only chemotherapy (Figure 3C).

#### 5.2.3 Mechanism-based subgroup analyses

Subgroup analyses for OS yielded results consistent with those observed in the overall population. Relative to CT, the PDL1+CT+Lur subgroup achieved the most prominent survival benefit (HR = 0.53, 95% CrI: 0.39–0.78), followed by PDL1+AA+CT (HR = 0.71, 95% CrI: 0.58–0.87). Similar trends were identified for PFS with more pronounced statistical effects: PDL1+CT+Lur showed the greatest progression-suppressing efficacy (HR = 0.37, 95% CrI: 0.29–0.47), with PDL1+AA+CT ranking second (HR = 0.38, 95% CrI: 0.32–0.46). Both subgroups demonstrated statistically significant superiority over all other categories. The subgroup findings further validated the robustness of the primary NMA outcomes (Figures 4A,B).

**FIGURE 4:**
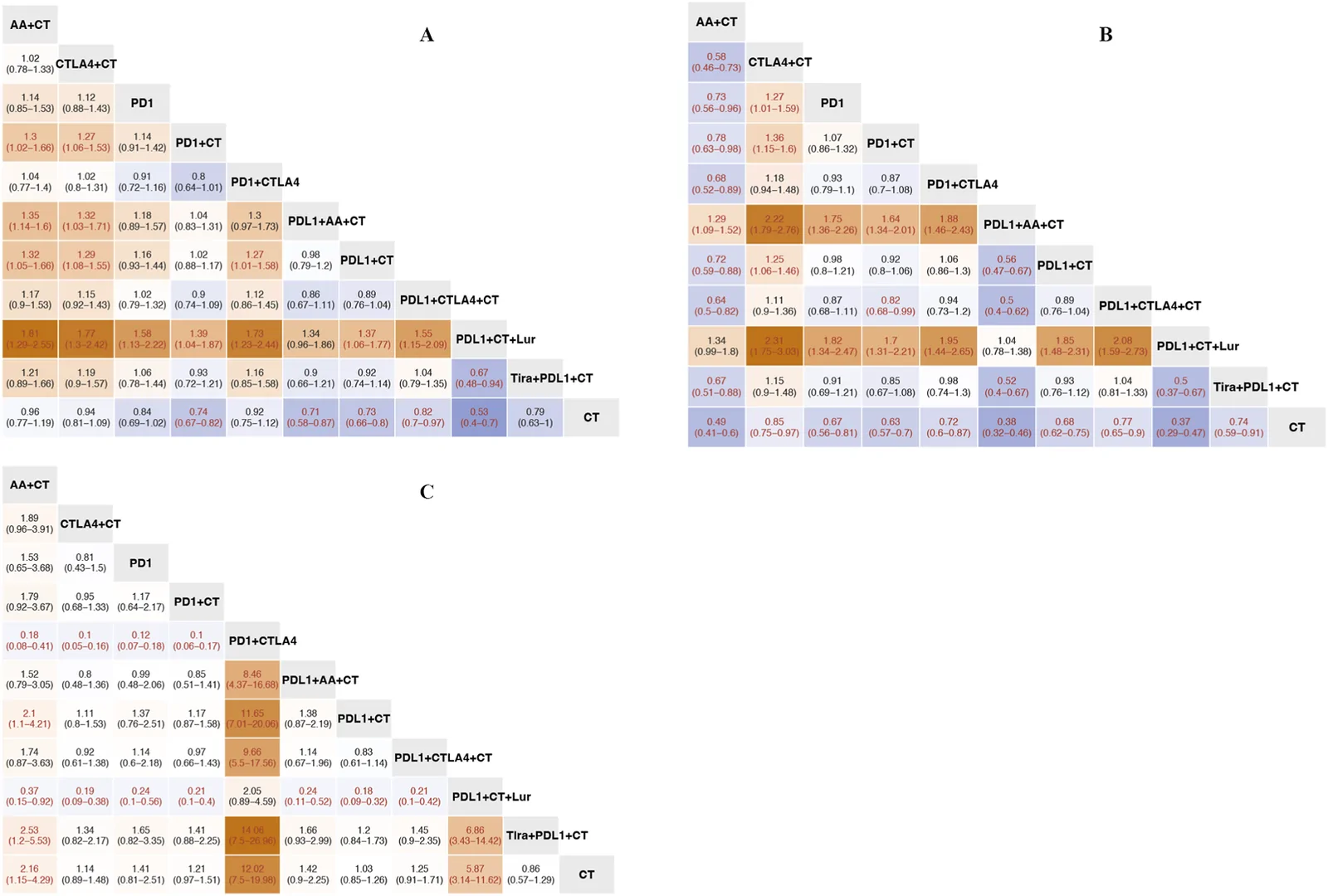
Efficacy and safety summaries from NMA in Pharmacological classification subgroup analysis: **(A)** OS, **(B)** PFS, and **(C)** AEs. The data in each cell represent the HR or OR values that compare treatment effects as defined by the columns and rows. The cells are color-coded according to the HR or OR values: values less than 1 are shaded blue, values greater than 1 are shaded orange, and cells with a value equal to 1 or missing data are filled with white. The important HRs (or ORs) and 95% CrIs are highlighted in red.

With respect to grade ≥3 AEs, the PD1+CTLA4 subgroup carried markedly higher toxicity risk than CT (OR = 12.02, 95% CrI: 7.50–19.98). The PDL1+CT+Lur subgroup (OR = 5.87, 95% CrI: 3.14–11.62) and AA+CT subgroup (OR = 2.16, 95% CrI: 1.15–4.29) exhibited the second and third highest risks of severe adverse events, respectively (Figure 4C).

## 6 SUCRA ranking analyses

SUCRA values for OS, PFS, and TRAEs of all eligible regimens are presented in Supplementary Figures S2, S3. It should be emphasized that SUCRA only reflects relative ranking probabilities of competing treatments, and minor numerical differences do not indicate clinical superiority. All ranking results were interpreted in combination with hazard ratios (HRs) and corresponding 95% credible intervals (CrIs).

For efficacy endpoints, Ate+CT+Lur yielded the highest SUCRA value of 93.39% for OS, followed by Ben+Anlo+CT (87.97%) and Ser+CT (87.65%). Multiple PD-L1 plus platinum-chemotherapy regimens showed moderate SUCRA values: Dur+CT (71.06%), Ade+CT (67.28%), and Pem+CT (47.88%). Platinum-only chemotherapy had a low SUCRA of 6.62%, while dual-immunotherapy regimens Nivo+Ipi (21.87%) and Ipi+CT (17.46%) occupied lower ranking positions. Similar ranking patterns were observed for PFS: Ben+Anlo+CT ranked first with a SUCRA of 99.43%, followed by Ate+CT+Lur (90.04%), Anlo+CT (87.27%), and Ser+CT (83.50%). Several dual-immune-blockade regimens obtained low SUCRA values ranging from 15.99% to 20.46%, including Ipi+CT, Dur+Tre+CT, and Tira+Ate+CT, whereas platinum-only chemotherapy reached merely 0.89%. Mechanism-stratified subgroup analyses demonstrated that PDL1+CT+Lur achieved top-ranked SUCRA values of 96.19% for OS and 92.41% for PFS. The overall ranking trends in subgroups were consistent with those in the full cohort, supporting the robustness of the primary ranking outcomes.

For safety endpoints, the interpretation of SUCRA was reversed: higher SUCRA values corresponded to lower relative risks of severe toxicities. Nivo+Ipi had the lowest SUCRA of 0.3%, indicating the highest relative risk of severe toxic events. Ate+CT+Lur (5.99%), Anlo+CT (15.44%), and Ben+Anlo+CT (21.22%) exhibited markedly lower SUCRA values relative to platinum-only chemotherapy (70.83%). Subgroup analyses stratified by therapeutic mechanisms were consistent with the overall findings. The dual PD-1 plus CTLA-4 immunotherapy regimen had the lowest SUCRA of 0.45%, indicating the poorest safety ranking. Regimens containing anti-angiogenic agents and maintenance therapy generally fell into inferior safety ranks, which may be attributable to additive vascular-related toxicities (hypertension, hemorrhage, proteinuria) induced by anti-angiogenic agents and cumulative adverse events resulting from long-term maintenance administration.

## 7 Sensitivity analyses

Sensitivity analysis was conducted by removing the only maintenance trial, CheckMate 451, to reassess all clinical outcomes. All HRs and 95% CrIs from the primary and sensitivity analyses are presented in Supplementary Table S3, while SUCRA-derived ranking results are visualized as a heatmap in Supplementary Figure S6. For PFS and OS, HR estimates and CrI boundaries for all chemotherapy-combined regimens were nearly identical between the primary and sensitivity analyses, and SUCRA rankings for most interventions remained consistent, demonstrating robust survival-related findings. For grade ≥3 AEs, minor shifts in CrI ranges were observed for most regimens, with only small fluctuations in SUCRA ranks and no reversal of statistical significance.

## 8 Discussion

This NMA systematically incorporated all published phase III RCTs investigating first-line immunotherapy induction and maintenance regimens for ES-SCLC. Eighteen interventions were quantitatively ranked across survival, anti-progression and safety endpoints via SUCRA values, and mechanism-stratified subgroup analyses were conducted to confirm the stability of the ranking results. The present study provides comprehensive quantitative evidence-based data to resolve clinical debates surrounding the optimal first-line treatment selection for treatment-naïve patients with ES-SCLC.

### 8.1 Efficacy stratification and underlying mechanisms

The OS and PFS rankings of the overall population demonstrated that two triple-drug induction-maintenance regimens, namely Ate+CT+Lur and Ben+Anlo+CT, ranked among the top two according to SUCRA probability values. Further subgroup analyses stratified by drug mechanisms revealed that the regimen of PD-L1 inhibitor combined with platinum-based chemotherapy followed by lurbinectedin maintenance (PDL1+CT+Lur) achieved the highest SUCRA value, which exerts synergistic anti-tumor effects. For the PDL1+CT+Lur regimen: on the one hand, PD-L1 inhibitors restore the infiltration of cytotoxic T cells within tumors and reverse tumor immune escape. Platinum-based chemotherapy induces immunogenic cell death (Patel and Minn, 2018), upregulates antigen presentation-related molecules, amplifies systemic anti-tumor immune responses, and positively remodels the tumor microenvironment (Wan et al., 2012; Gravett et al., 2018). On the other hand, lurbinectedin, a transcriptional inhibitor, directly triggers tumor cell apoptosis, modulates macrophage function and indirectly suppresses angiogenesis (Calles et al., 2025). It may mitigate acquired resistance to immune checkpoint inhibitors and improve OS and PFS. The synergistic anti-tumor activity of the Ben+Anlo+CT regimen is mainly mediated by anlotinib. As an anti-angiogenic agent, anlotinib remodels the tumor microenvironment to alleviate hypoxia-induced immunosuppression, facilitates the infiltration of neutrophils and mature dendritic cells, and reduces the proportions of myeloid-derived suppressor cells, regulatory T cells and tumor-associated macrophages (Roland et al., 2009; Yang et al., 2018), thereby generating synergistic anti-tumor efficacy with chemotherapy and benmelstobart.

Stratified comparisons indicated that combination regimens containing PD-L1 inhibitors yielded superior overall clinical benefits compared with PD-1 monotherapy, PD-1 plus CTLA-4 dual immunotherapy and platinum-based chemotherapy alone. Multiple network meta-analyses summarizing global phase III clinical trials including IMpower133, CASPIAN and KEYNOTE-604 confirmed that atezolizumab or durvalumab combined with platinum-etoposide significantly prolonged OS in ES-SCLC patients; pembrolizumab plus chemotherapy only improved PFS without statistically significant differences in OS; single CTLA-4 inhibitor combined with chemotherapy failed to extend overall survival and increased the incidence of immune-related adverse events (Wang et al., 2020; Wang et al., 2025). Molecular mechanisms account for such discrepancies: PD-L1 monoclonal antibodies block PD-L1 expressed on both tumor cells and dendritic cells while preserving homeostatic PD-L2 signaling on T cells and macrophages to avoid excessive immune activation, which is more compatible with the severely immunosuppressive microenvironment of SCLC. By contrast, CTLA-4 inhibitors only act on the initial stage of immune activation, fail to sustainably reverse local tumor immune escape, and tend to induce severe systemic immune toxicities, shortening the window of continuous clinical administration (Zhang and Cheng, 2023). The cumulative probability rankings of SUCRA values in this study were consistent with conclusions from existing evidence-based reviews, further supporting that first-line PD-L1 combination induction followed by maintenance therapy represents a superior therapeutic strategy for ES-SCLC.

This study simultaneously compared two distinct therapeutic modalities: synchronous chemoimmunotherapy induction and PD-1 maintenance without induction chemotherapy. The CheckMate 451 trial enrolled ES-SCLC patients who achieved partial response or stable disease after four cycles of single-agent platinum chemotherapy, who were subsequently assigned to three arms: PD-1 monotherapy maintenance, PD-1 plus CTLA-4 dual immunotherapy maintenance, and placebo. The primary endpoint was negative: PD-1 maintenance regimens with or without dual immunotherapy without synchronous induction chemotherapy failed to significantly prolong OS. The SUCRA rankings of our study validated this result from the efficacy perspective, where all synchronous chemoimmunotherapy induction regimens exhibited markedly higher SUCRA values for OS and PFS than the PD-1 maintenance group without induction. Mechanistically, synchronous administration of chemotherapy and immunotherapy in treatment-naive patients with high tumor burden sufficiently induces immunogenic cell death, releases abundant tumor neoantigens and establishes long-lasting anti-tumor memory T cell responses. In contrast, sequential immunotherapy maintenance after tumor shrinkage by chemotherapy reduces neoantigen abundance, aggravates immunosuppression within the tumor microenvironment, and leads to inadequate activation of anti-tumor immunity (Li et al., 2025; Bishara et al., 2026).

### 8.2 Safety profiles and risk considerations of different treatment modalities

Although triple-drug induction-maintenance regimens deliver prominent survival benefits, their generally low SUCRA values for grade ≥3 adverse events suggest substantially elevated risks of severe toxicities. The most common severe adverse events of Ate+CT+Lur and Ben+Anlo+CT included myelosuppression, fatigue, gastrointestinal injuries and hypertension (Lu and Ades, 2004; Paz-Ares et al., 2025a). Among them, Ate+CT+Lur produced the most remarkable survival gain accompanied by the most prominent superimposed toxic effects. Prior to initiating such triple-drug regimens, clinicians should comprehensively evaluate patients’ baseline bone marrow reserve, physical status and gastrointestinal tolerance. Regular radiological examinations and laboratory tests are required throughout maintenance therapy for early identification and timely intervention of severe adverse reactions.

Subgroup safety analyses stratified by therapeutic mechanisms revealed that all regimens incorporating anti-angiogenic agents and long-term maintenance therapy ranked poorly in safety outcomes. Anti-angiogenic drugs induce superimposed vascular-related toxicities such as hypertension, hemorrhage and proteinuria, and prolonged maintenance administration further accumulates adverse reactions, substantially increasing the burden of severe toxicities.

Dual immunotherapy represented by Nivo+Ipi displayed marked safety defects: limited efficacy accompanied by SUCRA values close to zero for grade ≥3 adverse events. Dual blockade of PD-1 and CTLA-4 excessively amplifies systemic immune responses and breaks peripheral immune tolerance, substantially elevating the risks of multi-organ severe immune-related adverse events including severe pneumonia, fulminant hepatitis and endocrine dysfunction (Postow et al., 2018; Wang et al., 2023). Data from the CheckMate 451 trial also verified that adding ipilimumab to nivolumab maintenance failed to improve OS while significantly increasing the incidence of severe toxicities. Accordingly, PD-1/CTLA-4 dual immunotherapy induction and maintenance might not be favoured for routine first-line usage for ES-SCLC, and may only be cautiously adopted for young patients with excellent physical status after full informed consent.

Distinct safety gaps exist between synchronous induction regimens and PD-1 maintenance without induction: the synchronous chemoimmunotherapy regimen PD1+CT yielded a favorable safety SUCRA value of 55.9%; the SUCRA value of PD-1 monotherapy without induction was merely 45.98%; the dual immunotherapy regimen PD1+CTLA4 without synchronous chemotherapy had thelowest SUCRA value of 0.46% across all stratified regimens, indicating the worst safety profile. Although PD-1 maintenance without induction avoids platinum-induced myelosuppression, it carries higher risks of persistent multi-organ immune-related adverse events. Combining clinical guidelines, CheckMate 451 data and quantitative SUCRA stratification results of this study, treatment-naive ES-SCLC patients with intact organ function tolerance may be preferentially considered for synchronous chemoimmunotherapy induction.

### 8.3 Maintenance therapy and future perspectives

Multiple existing reviews have proposed that maintenance therapy represents the core direction for optimizing first-line regimens of ES-SCLC (Qiang et al., 2024; Paz-Ares et al., 2025b; Shalata et al., 2025). Maintenance strategies enrolled in trials included three categories: single-agent ICIs maintenance, dual immunotherapy maintenance, and immunotherapy combined with anti-angiogenic maintenance. Comparative analyses demonstrated that dual immunotherapy maintenance fails to improve efficacy while significantly increasing severe toxicities; single-agent ICIs maintenance yields modest efficacy gains relative to chemotherapy alone with lower toxicities; anti-angiogenic combination maintenance improves survival outcomes yet aggravates the burden of adverse reactions simultaneously.

Consistent with the above observations, our NMA SUCRA rankings further quantified this efficacy-safety trade-off across maintenance-containing regimens. Triplet strategies consisting of PD-L1-based immunochemotherapy induction followed by maintenance therapy achieved favorable survival SUCRA ranks, whereas regimens incorporating anti-angiogenic agents attained competitive survival performance but occupied inferior positions in safety SUCRA ranking. From a mechanistic perspective, effective induction immunochemotherapy can promote tumour-associated antigen release and activate intratumoural cytotoxic T-cell responses. Subsequent maintenance treatment may sustain anti-tumour immune surveillance, suppress early tumour relapse and delay the onset of acquired resistance. Nevertheless, anti-angiogenic agents add vascular-related toxicities such as hypertension, proteinuria and haemorrhage; long-term maintenance further accumulates adverse events, which may offset survival benefits and impair patients’ treatment tolerance.

Several clinical questions remain unresolved: the optimal timing to introduce anti-angiogenic agents (induction versus maintenance phase) is unclear, and unified criteria for precise targeted agent selection among different patient subgroups are lacking. These aspects can be regarded as core entry points for subsequent clinical trials and real-world studies. Future prospective trial designs should strive to separate and report endpoints for induction and maintenance phases individually, so as to precisely quantify the incremental benefit contributed by maintenance components. More head-to-head comparisons among diverse maintenance modalities rather than merely maintenance versus observation are urgently required. Biomarker-stratified randomisation designs are encouraged to screen populations most likely to benefit from specific maintenance regimens and balance survival gain and toxic risk. Additionally, long-term real-world evidence is needed to externally validate the SUCRA-derived ranking conclusions, especially the long-term safety profile of continuous maintenance administration in ES-SCLC patients.

## 9 Study limitations

Several limitations of the present study should be acknowledged. First, original studies only reported combined efficacy data covering the full induction-maintenance period, without separated survival and safety outcomes for each stage. Accordingly, we failed to quantify the independent anti-tumor benefits of induction and maintenance therapy, which partially reduces the evidence strength of our stratified treatment suggestions. Second, subgroup analyses based on age, ECOG score and brain metastasis were not conducted, as trials adopted inconsistent stratification cutoffs and lacked complete combinable quantitative indicators. Third, only grade ≥3 TRAEs were adopted as the safety endpoint, without separate grading of organ-specific immune-related adverse events, preventing cross-regimen comparison of organ-targeted toxicity profiles. Further large-scale prospective RCTs reporting phase-separated endpoints and standardized real-world cohorts are required to optimize staged individualized therapeutic strategies.

## 10 Conclusion

PD-L1 inhibitor combined with platinum-based chemotherapy together with anti-angiogenic agents or lurbinectedin conferred optimal survival benefits, yet toxicity was correspondingly augmented. This study provides valuable insights into therapeutic drug selection for ES-SCLC.

## Data Availability

The original contributions presented in the study are included in the article/Supplementary Material, further inquiries can be directed to the corresponding author.
