# Supplementary material for: Efficacy and safety of first-line immunotherapy-related induction and maintenance therapy for extensive-stage small cell lung cancer: a systematic review and Bayesian network meta-analysis
Source: Front Pharmacol. 2026 Sep 15;17:1944817. doi: 10.3389/fphar.2026.1944817 (PMC13619831; doi:10.3389/fphar.2026.1944817)
Supplement: Supplementary file 1 [file Supplementaryfile1.docx]

**Efficacy and Safety of First-line Immunotherapy-related Induction and Maintenance Therapy for Extensive-Stage Small Cell Lung Cancer: A Systematic Review and Bayesian Network Meta-Analysis**

Table S1 Literature search strategy

((((("Small Cell Lung Carcinoma"[Mesh]/mh OR "small cell lung cancer" OR SCLC OR ES-SCLC OR "oat cell carcinoma") AND ("extensive-stage" OR "extensive disease" OR extensive)) AND ("Immunotherapy"[Mesh]/mh OR immunotherap OR "immune checkpoint inhibitor" OR ICI OR chemoimmunotherap OR PD-1 OR PD-L1 OR CTLA-4 OR atezolizumab OR durvalumab OR pembrolizumab OR nivolumab OR ipilimumab OR tiragolumab OR sintilimab OR toripalimab OR camrelizumab OR tislelizumab OR adebrelimab OR serplulimab OR socazolimab OR bevacizumab OR anlotinib)) AND (("first-line" OR "treatment-naive" OR "previously untreated") OR (induction OR maintenance OR consolidation))) AND (randomized controlled trial[pt] OR controlled clinical trial[pt] OR randomized OR randomised)) NOT review[Filter] NOT animals[Filter] NOT case report[Filter])

Supplementary Material

|  |   Figure S1  Risk-of-bias evaluation of eligible randomized trials using the Cochrane revised risk-of-bias tool 2 (RoB 2). Left panel: Domain-specific and overall risk-of-bias judgments for each included trial. Right panel: Bar graph showing the proportional distribution of studies judged as low risk (green) or some concerns (yellow) across five bias domains (randomization process, deviations from intended interventions, missing outcome data, outcome measurement, selection of reported results) and the overall risk of bias. |
| --- | --- |

Figure S2 SUCRA Ranking Curves Across Three Clinical Endpoints

A

OS

B

PFS

C

≥3AE

Figure S3

Rankograms illustrating rank probabilities of all first-line regimens in the full overall cohort of treatment-naïve ES-SCLC patients derived from Bayesian network meta-analysis. For efficacy endpoints (A, B), rank 1 represents superior therapeutic efficacy; for safety endpoint (C), rank 1 indicates the lowest risk of severe adverse events. The horizontal axis lists all included treatment regimens, and the vertical axis denotes the probability of each regimen occupying a specific rank, visualized by stacked vertical bars.

OS

A

PFS

B

≥3AE

C

Figure S4 Rankograms illustrating rank probabilities of all first-line regimens in Pharmacological classification subgroup analysis of treatment-naïve ES-SCLC patients derived from Bayesian network meta-analysis. For efficacy endpoints (A, B), rank 1 represents superior therapeutic efficacy; for safety endpoint (C), rank 1 indicates the lowest risk of severe adverse events. The horizontal axis lists all included treatment regimens, and the vertical axis denotes the probability of each regimen occupying a specific rank, visualized by stacked vertical bars.

|   A  OS |   PFS |   ≥3AE |
| --- | --- | --- |
|   B  OS |   PFS |   ≥3AE |
| Figure S5. Bayesian network meta-analysis forest plot of HR/OR (95% CrI) for all treatment arms versus CT and Ate+CT/PDL1+CT. HR/OR = 1 is the null effect line; HR/OR > 1 means inferior efficacy compared with reference regimen. (A) All therapeutic regimens in the overall patient cohort (B) Pharmacological classification subgroup analysis. | | |

| **Table S2 Comparisons of the fit of consistency and inconsistency models using deviance information criteria (DIC)** | | | |
| --- | --- | --- | --- |
| Outcomes | **Model** | | |
|  | **Consistency Fixed** | **Consistency Random** | **Inconsistency** |
| Progression-free survival | 33.96 | 34.02 | 33.96 |
| Overall survival | 33.99 | 34.04 | 34.07 |
| Grade ≥3 adverse events | 62.41 | 62.52 | 62.45 |

Figure S6 The Brooks-Gelman-Rubin diagnostic and the density trace plot.

A. OS

B. PFS

C. ≥3AE
